# Retraction: Downsizing and purchases of psychotropic drugs: A longitudinal study of stayers, changers and unemployed

**DOI:** 10.1371/journal.pone.0285004

**Published:** 2023-04-20

**Authors:** Sandra Blomqvist, Kristina Alexanderson, Jussi Vahtera, Hugo Westerlund, Linda L. Magnusson Hanson

After this article [1] was published, the authors identified an error in the script used to stratify people exposed to downsizing that led to misclassification of some individuals as Stayers instead of Changers. Re-analyses indicated that this issue affected subgroup classification and key aspects of the article’s results and conclusions, although the overall conclusion trended in the same direction as was reported in [1]. Therefore, the authors retract this article. We plan to publish an updated version of this work in which the errors have been corrected.

All authors agreed with the retraction and apologize for the issues with the published article [1].
